# Increase in prefrontal cortex oxygenation during static muscular endurance performance is modulated by self-regulation strategies

**DOI:** 10.1038/s41598-018-34009-2

**Published:** 2018-10-25

**Authors:** Wanja Wolff, Maik Bieleke, Anna Hirsch, Christian Wienbruch, Peter M. Gollwitzer, Julia Schüler

**Affiliations:** 1 Institute of Sport Sciences, Sport Psychology, University of Konstanz, Konstanz, Germany; 2 Department of Psychology, University of Konstanz, Konstanz, Germany; 3 Graduate School of Decision Sciences, University of Konstanz, Konstanz, Germany; 4 Department of Psychology, New York University, New York, USA

## Abstract

Enduring physical strain is an important ability and prototypically required in athletic activities. However, little is known about the psychological determinants of endurance performance and their underlying neural mechanisms. Here, we investigated self-regulation as one such factor. We recruited 60 participants who hold intertwined rings for as long as possible while avoiding contacts between them, either with a goal intention or an implementation intention to perform well. Performance was measured in terms of time-to-failure and contact errors. Additionally, we repeatedly assessed ratings of perceived exertion (RPE) and pain (RPP) and used functional near-infrared spectroscopy (fNIRS) to continuously monitor cerebral oxygenation in dorsal and ventral parts of the lateral prefrontal cortex (LPFC), brain regions associated with effortful attentional control and response inhibition, respectively. Performance, RPE and RPP were similar in the goal and the implementation intention condition. LPFC activity increased over time, but its activation level was generally lower in the implementation intention condition. Both effects were particularly pronounced in the dorsal LPFC. Moreover, the balance between effortful and more automatic regulation seems to differ between self-regulation strategies. Our results indicate that self-regulation plays an important role in endurance performance and that self-regulatory processes during endurance performance might be reflected in LPFC activation.

## Introduction

The ability to endure physical strain over extended periods of time is an important human capacity, required in diverse work settings (e.g., hospitals, factories) and most prototypically during athletic activities (e.g., running, cycling). A large body of research has aimed at identifying the limits of endurance performance, based on the assumption that people disengage from an endurance task primarily because their physiological resources are depleted (e.g.^1–4^). This assumption has been challenged by research showing that individuals feel exhausted and disengage from a straining activity even though their physiological resources would allow them to continue (e.g.^5,6^).This suggests that psychological (rather than exclusively physiological) factors too play an important role in determining the limits of endurance performance. However, little attention has been paid to such psychological factors and their underlying neural mechanisms so far^7,8^. In the present research, we investigated self-regulation as one potential factor, focusing on behavioral effects and cerebral correlates.

Endurance performance is associated with various aversive sensations like muscle pain, feelings of exertion, fatigue, and the urge to quit^7^. Successful performance should therefore depend on whether individuals are motivated to and capable of investing the effort required for dealing with these sensations. In line with this reasoning, the psychobiological model of exercise tolerance^9,10^ assumes that people consciously decide to disengage form a strenuous task as soon as continued effort seems no longer justified or possible. Accordingly, endurance performance is not ultimately limited by physiological resource depletion; exhaustion and task termination should instead reflect a self-regulated decision to disengage^11^. From this perspective, endurance performance can be understood as a task with incremental self-regulatory demands: As maintaining certain levels of performance becomes increasingly difficult (i.e., the perception of effort rises), the self-regulatory demands faced by the athlete rise as well (e.g., suppressing the impulse to quit).

Attesting to this assumption, experimental manipulations of self-regulation can indeed affect exercise-related sensations (e.g., perceived effort and pain^12^) and endurance performance (e.g.^13,14^). On the neural level, corresponding evidence comes from the finding that strenuous cardiovascular endurance performance is accompanied by increasing activation in the prefrontal cortex (PFC; systematic review by^15^). Although this finding is usually discussed in the light of physiological changes induced by the task, the PFC also plays a critical role for enabling people to effectively self-regulate their behavior (e.g.^16,17^). Due to its anatomical position the PFC is ideally suited for playing a critical role in human self-regulation: it receives input from “virtually all sensory systems, with cortical and subcortical motor system structures, and with limbic and midbrain structures involved in affect, memory, and reward”^18^ (p. 174). The PFC is also strongly connected with motor system structures associated with the voluntary control of behavior^18^. Within the PFC, different areas appear to be involved in distinct facets of self-regulation (e.g.^18,19^). For instance, dorsolateral areas are involved in top-down attention control, whereas ventrolateral areas are important for response inhibition^19^, facets of self-regulation that seem important in an endurance task: When peak performance is required (e.g., at the Olympic Games), minor lapses in attention (getting distracted by a cheering crowd) or failure to inhibit an undesired response (e.g., urge to match the higher pace of an opponent) can decide between winning and losing^20^.

It is plausible that one limiting factor in endurance performance is the efficacy to self-regulate behavior. If this is the case, performance should benefit from effective self-regulation strategies. Indeed, a range of such strategies has been shown to enhance athletic performance – such as goal setting^21,22^, self-talk^23,24^, attentional control^25,26^, or combinations of them in the form of psychological skill training packages (PST)^27^. Whether and how these strategies might specifically facilitate endurance performance is, however, not well established^28^. In the present research, we turned to the brief and effective self-regulation strategy of forming implementation intentions^29^. It involves mentally linking critical obstacles or opportunities to act with goal-directed behaviors: “If I encounter Situation S, then I will perform Behavior B!” As a consequence, situations specified in the if-part become cognitively more accessible and associatively linked with goal-directed behaviors specified in the then-part^30^. Thus, forming implementation intentions enables people to automatically initiate and maintain goal-directed behaviors when encountering the specified critical situations – an efficient bottom-up form of action control in contrast to the more resource-demanding and yet less effective top-down action control with goal intentions (e.g., “I want to perform behavior B!”)^31^. A large body of literature suggests that forming implementation intentions helps people better attain their goals across various domains^32^. However, the question of whether implementation intentions facilitate endurance performance more effectively than goal intentions has only recently been addressed. The existing evidence suggests that implementation intentions can regulate exercise-related sensations in endurance tasks but their effects on performance have so far been inconsistent^20^.

An important characteristic of implementation intentions is that they automate goal-directed behavior. This notion of strategic automaticity is corroborated by neuroscientific evidence on the temporal distribution and spatial location of implementation intention effects in the brain^33^. Implementation intentions have been demonstrated to modulate event-related potential components like the P100, N170, and P300^34–37^, indicators of early information processing that are otherwise beyond voluntary control. Moreover, implementation intentions evoke activity in brain areas associated with automatic, bottom-up action control, whereas goal intentions recruit brain areas associated with deliberative, top-down action control^38,39^. For instance, in a study using functional magnetic resonance imaging, Gilbert *et al*.^39^ found that forming implementation intentions was associated with greater activity in the medial rostral PFC, while goal intentions were associated with activity in the lateral PFC (LPFC). Similarly, Hallam *et al*.^38^ showed that goal intentions influenced activity in the dorsolateral PFC as well as areas outside the prefrontal cortex, whereas implementation intentions activated the orbitofrontal cortex. These findings are in line with the notion that the PFC is differentially involved in the self-regulation of behavior.

## Study Aim

Taken together, self-regulation seems to be an important psychological factor in the context of endurance performance. On a cerebral level, this is likely associated with activity in the LPFC, a brain region that has been implicated in self-regulation. Accordingly, this study aimed at investigating the role of self-regulation on performance and cerebral oxygenation in a static muscular endurance task. In this task, participants held two intertwined rings for as long as possible while avoiding contacts between the rings. Performing this task should be accompanied by increasing sensations of effort and pain that must be effectively regulated. We expected that forming implementation intentions permits a more automatic and effective regulation of aversive sensations during the task compared to forming mere goal intentions, ultimately leading to reduced perceptions of these sensations and better performance. We additionally monitored cerebral oxygenation in the LPFC with functional near-infrared spectroscopy (fNIRS) – a technique to monitor cerebral oxygenation that is particularly promising for more naturalistic experiments^40^ – to investigate brain activity in a region that is tightly linked to effortful, deliberate cognitive control. As dorsal and ventral parts of the LPFC have been observed to orchestrate different facets of self-regulation, we analyzed them as separate regions of interest (ROI). Overall, we expected LPFC oxygenation to increase as a function of time on task, thereby reflecting incremental self-regulatory demands. Moreover, we expected that implementation intention participants generate lower levels of LPFC activation than goal intention participants, reflecting the expected reduced requirement for effortful control. An exploratory question was whether this predicted activation pattern would differ between dorsal and ventral areas of the LPFC.

## Methods

### Participants and Design

Sixty female students took part in the study (age: *M* = 22.4, *SD* = 3.3) for a monetary compensation of 12 Euro or course credit. Implementation intentions have medium-to-large effects (*d* = 0.65, for a meta-analysis, please see^30^), which allows us to detect differences between conditions in two-sample independent *t*-tests at 80% power. Participants were randomly assigned to a goal (*n* = 30) or an implementation intention condition (*n* = 30). One might argue that the effects of self-regulation strategies should ideally be investigated in counterbalanced within-participant designs. Unfortunately, however, it is impossible to prevent carry-over effects of forming implementation intentions on a subsequent goal intention condition, invalidating any comparisons between the conditions. Accordingly, prior research on the behavioral effects^30,41^ and the neurophysiological correlates of implementation intentions^34,35,38^ has used between-participant designs and we adhered to this common standard. Written informed consent was obtained from all participants prior to starting the experiment. The experimental protocol and all methods that were employed were approved by the Ethics Committee at the University of Konstanz (approval #24/2016).

The participants reported to engage in *M* = 2.4 h (*SD* = 2.1, *Min* = 0, *Max* = 12.5) of sport per week, 20.1% of which they ascribed to strength training. Participants who reported a main sport (87.7%) had performed it for an average of *M* = 5.0 years (*SD* = 4.9). No differences between the goal and the implementation intention condition evinced regarding the number of hours of sport per week, *p* = 0.914, or regarding the average duration of performing the main sport, *p* = 0.789. Because the task involved holding weights, only participants with no current or recent injuries of shoulders, arms, or the back were eligible for participation. To reduce the large variance that is typically observed in strenuous time-to-failure tasks, we only sampled female participants to avoid additional between-gender variance^12,42^. Participants indicated whether they had engaged in straining exercise (7 participants in each condition answered “yes”) or consumed alcohol (4 goal and 3 implementation intention participants answered “yes”) the day before, and whether they consumed caffeine in the two hours prior to the experiment (1 goal intention participant answered “yes”). The conditions did not differ significantly in their answers to any of these questions, all three *p*^1^s > 0.3.

### Measures and Apparatus

#### Static muscular endurance task

**Figure 1:**
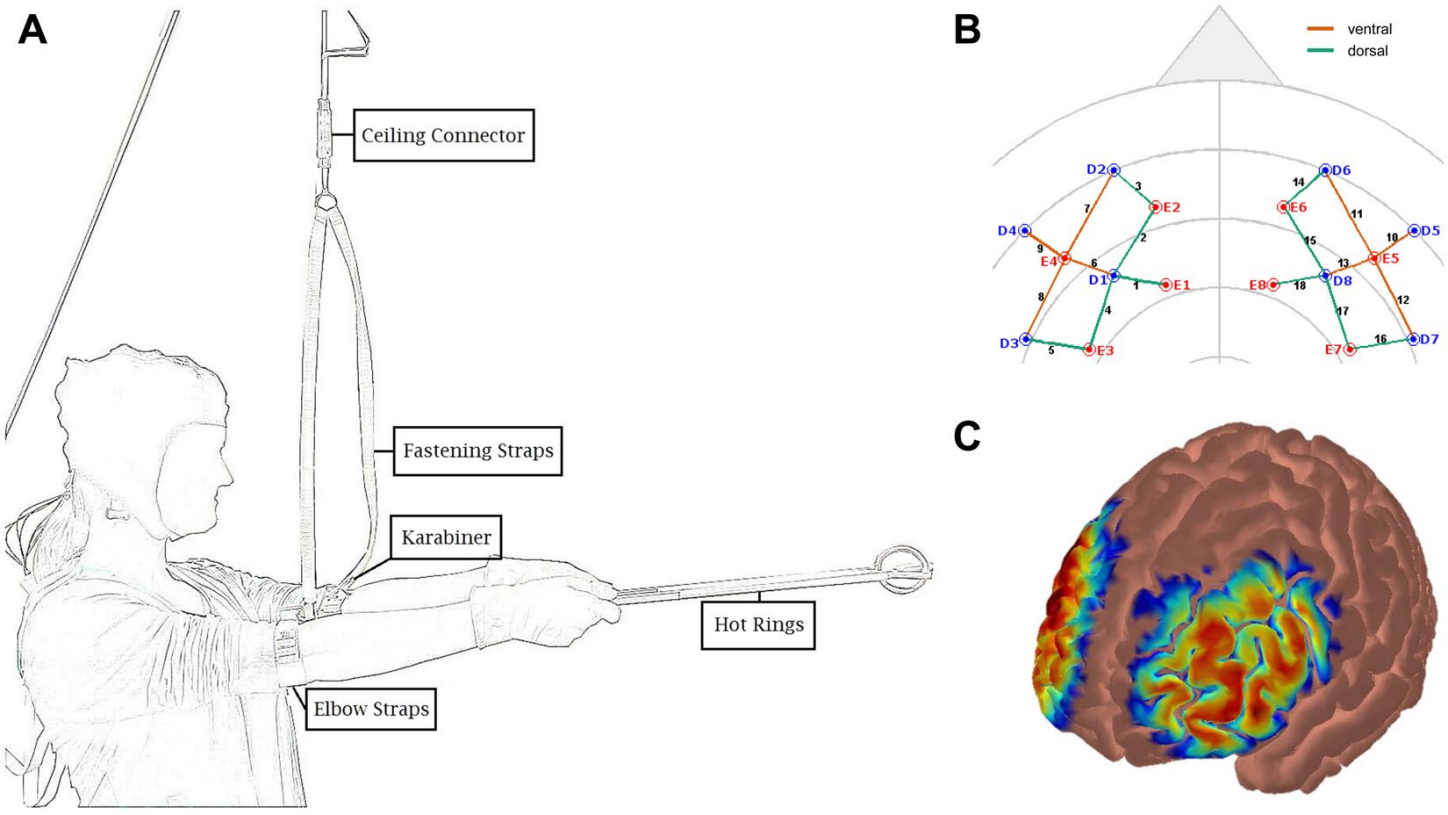
Schematic illustration of the “hot rings task” (HRT, **A**). For the fNIRS measurement, emitters (E) and detectors (D) were positioned according to the international 5/10 system: E1 at F1, E2 at AF3, E3 at FC3, E4 at F5, D1 at F3, D2 at AF7, D3 at FC5, D4 at F7, E5 at F6, E6 at AF4, E7 at FC4, E8 at F2, D5 at F8, D6 at AF8, D7 FC6, and D8 at F4. This montage was designed to measure activity over dorsal (Emitter-detector combinations: E1_D1, E2_D1, E3_D1, E6_D8, E7_D8, E8_D8, E2_D2, E3_D3, E6_D6) and ventral (Emitter-detector combinations: E4_D1, E4_D2, E4_D3, E5_D5, E5_D6, E5_D8) areas of the LPFC (**B**). The sensitivity profile (Atlas Viewer^76^) of the montage indicates that that the chosen optode placements capture the LPFC reasonably well. It represents Monte Carlo random walks of 1e^7^ photons (per optode) migrating through a standard atlas (Colin27, **C**).

To measure static muscular endurance performance, we used the “hot rings task” (HRT) introduced by Bieleke and Wolff^12^. In the HRT, participants hold two aluminum bars connected by intertwined rings for as long (time-to-failure) and with as few contacts between the rings (errors) as possible. They stand in an upright position with their arms outstretched to form a 90° angle with their torso. To reliably measure time-to-failure and errors, participants’ arms are strapped into a holding device connected to the ceiling of the laboratory via a connector element (see Fig. 1A). The holding device is adjusted to participants’ height prior to the task with the connector element locked to prevent pre-task exertion. At the beginning of the task, the connector element is unlocked and therefore unplugs as soon as participants’ arms drop below the preset 90° angle. Ring contacts are continuously measured at 50 Hz with a recording box connected to the aluminum bars.

The HRT has a unique advantage over other performance measures used in self-regulation research (e.g., hand dynamometers^43^): it not only allows to measure performance quantity but also provides a built-in measure for performance quality. This additional measure is particularly important in a sport context because quantitative performance increases at the cost of decreasing performance quality might lead to errors and injuries (e.g.^44^).

#### Assessment of cerebral oxygenation

An 8 emitter + 8 detector multichannel continuous-wave fNIRS imaging system (NIRSport, NIRx Medical Technologies LLC, NY, USA) was used to monitor changes in cerebral concentrations during the HRT. NIR light was emitted in two wavelengths (760 nm and 850 nm) at a sampling rate of 7.81 Hz. To capture fluctuations in LPFC activation, two 4 emitter +4 detector arrays were bilaterally positioned over scalp sites corresponding to the LPFC (see Fig. 1B). Optode placement was done according to the international 5/10 system^45^. Channels of interest were emitter-detector pairs with 3 mm separation. This resulted in nine channels on the left (channels 1–9) and in nine channels on the right (channels 10–18) hemisphere. Channels 9, 12, and 16 had to be excluded from the analyses due to detector malfunction. The probes were fixated in a custom-made stretchy fabric NIRScap (EASYCAP GmbH, Herrsching, Germany) with an interoptode distance of 30 mm. The NIRScaps for optode placement were available in three different sizes (head circumferences of 54, 56, and 58 cm) and suitable for all our participants. A retaining overcap (EASYCAP GmbH, Herrsching, Germany) was placed over the NIRScap in order to exert pressure evenly onto the probes, which enhances the contact between the probe tips and the scalp, stabilizes the setup against motion artifacts, and shields the optodes from ambient light.

#### Ratings of perceived exertion (RPE) and pain

While performing the HRT, participants were repeatedly prompted to rate their perceived exertion (RPE) and pain using the category ratio 10 (CR10) scale by Borg^46,47^. The CR10 scale is “a general intensity scale that can be used to estimate most kinds of perceptual intensities”^46^ (p. 15). It can be used in various settings and for different purposes (i.e., studies in medicine and sports). Participants were provided with a definition of effort (“the conscious sensation of how hard, heavy, and strenuous a physical task is;”^48^) to clearly distinguish RPE from pain as suggested by Pageaux^49^. The two scales ranged from 0 (“nothing at all”) to 10 (“maximal”) and were printed on separate sheets of paper placed on a wall in front of the participants. To avoid ceiling effects, the CR10 also includes the option for ratings greater than 10 (“even more than max”) because during exertion some subjects might realize they can tolerate even higher levels of effort or pain^46^.

### Procedure

Each session was carried out by two researchers who started the session by preparing the fNIRS measurement and adjusting the HRT holding device. They instructed participants about the task and the CR10 scale and provided a demonstration trial in which participants deliberately lowered their arms below the preset 90° angle to become familiar with the sensitivity of the connector element. This was followed by a 2 min baseline fNIRS measurement.

#### Self-regulation strategies

Upon the baseline measure, participants in the goal condition rehearsed the goal for the endurance task (*“The task is to persist for as long as possible while avoiding contacts between the rings!”*). Participants in the implementation intention condition adopted the same goal (*“I want to persist for as long as possible while avoiding contacts between the rings!”*) and furnished it with an implementation intention (*“If the task becomes too strenuous for me, then I will ignore the strain and tell myself: ‘Keep going!”*). An experimenter read these instructions aloud and participants repeated it verbally.

#### Endurance task

After they had received their self-regulation strategies, participants were strapped into the HRT holding device and received the aluminum bars. The two experimenters unlocked the connector element, switched on the recording box, and started a timer to measure time-to-failure. The experimenters did not interact with participants during the task and remained outside their field of vision. The prompts for RPE and pain ratings were prepared sound recordings^50^ played by the computer every forty seconds with a jitter of plus-minus ten seconds. The prompts for RPE and pain were separated by ten seconds. Answers were documented by the experimenters. The task ended as soon as the connector element unplugged.

#### Final questionnaire

The experiment ended with a final questionnaire assessing the motivation to perform well with six items (Cronbach’s α = 0.80; e.g., “It was important for me to persist for as long as possible in the endurance task”) to be answered on Likert scales (1: *does not apply*, 7: *fully applies*). Finally, participants answered a question about current exhaustion and demographic questions (e.g., gender, age, physical activity).

### Data Analytic Strategy

Comparisons between conditions were made with *t*-tests for dependent variables measured only once (e.g., time-to-failure, performance motivation). Other variables were measured repeatedly during the task and were thus subjected to mixed-effect ANOVAs. As the repeated measures were assessed at different frequencies and participants varied considerably in their time-to-failure, we divided time-to-failure into 10% intervals. This yielded between 2 and 10 observations per participant. Behavioral variables (i.e., errors, effort, and pain) were analyzed in 2-between (Condition: goal intention, implementation intention)×10-within (Time-to-Failure: [0–10%], (10–20%], …, (90–100%]) ANOVAs. Estimates of cerebral oxygenation (i.e., oxygenated (O_2_Hb) and de-oxygenated (HHb) hemoglobin concentration) were subjected to 2-between (Condition: goal intention, implementation intention)×10-within (Time-to-Failure: [0–10%], (10–20%], …, (90–100%])×2-within (Region of Interest (ROI): dorsal LPFC, ventral LPFC) ANCOVAs with average baseline concentration as covariate.

Data analysis was conducted using the statistical software R^51^. ANOVAs were estimated with the lme4 package (version 1.1-14^52^) using the Satterthwaite approximation of lmerTest (version 2.0-33^53^). Significant interaction effects were followed up by Bonferroni-adjusted contrasts using lsmeans (version 2.27–60^54^). Plots were created with ggplot2 (version 2.2.1^55^).

#### fNIRS preprocessing

fNIRS data were preprocessed using HOMER2^56^ (MathWorks Inc., 2016). For each participant, the *enPruneChannels* function was used to remove channels when the signal was too weak or too strong. Then, optical intensity was converted to optical density using the *Intensity_to_OD* function. To remove motion artifacts, the *Wavelet_Motion_Correction* was run with an IQR of 1.0^57^. This method entails a discrete wavelet transformation on the data and has been shown to be efficient in recovering the hemodynamic response function^58^ (HRF). Then, data were low pass filtered (0.5 Hz) and converted to oxy- and deoxyhemoglobin with the modified Beer-Lambert law^59^. For this conversion, differential path length factors of 7.3 (for 760 nm) and 6.4 (for 850 nm) were chosen^60^.

## Results

### Behavioral Data

#### Task motivation

Participants were motivated to perform well on the task (*M* = 6.1, *SD* = 0.7), similarly in the goal (*M* = 6.2, *SD* = 0.6) and the implementation intention condition (*M* = 6.0, *SD* = 0.7), *t*(58) = 1.27, *p* = 0.208.

#### Task performance

**Figure 2:**
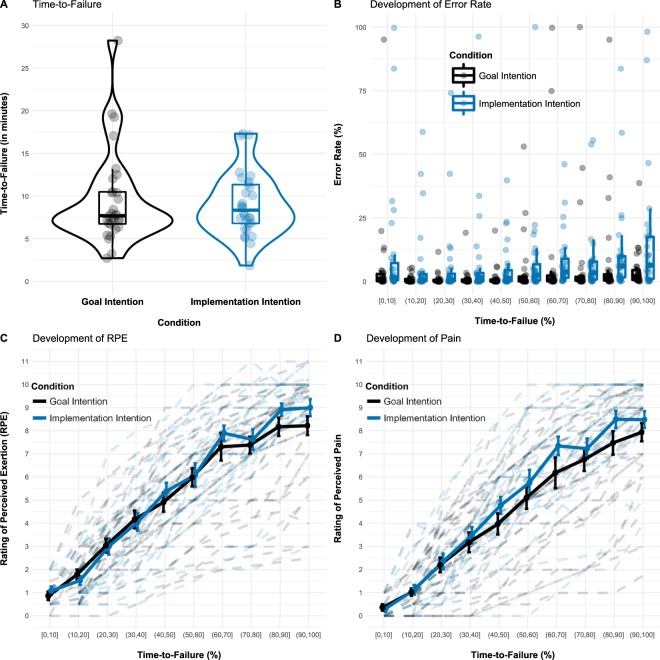
Behavioral results as a function of Condition and Time-to-Failure. Participants in both conditions persisted similarly long in the static muscular endurance task (**A**) and while error rates were generally low, implementation intention participants tended to make more errors than goal intention participants (**B**). Both RPE (**C**) and perceived pain (**D**) increased over time with no reliable differences between conditions. Error bars in (**B,C**) represent standard errors of the mean.

Time-to-failure varied between 1.9 to 28.2 minutes with an average of *M* = 9.3 minutes (*SD* = 4.6), consistent with a previous study^12^. No differences evinced between the goal (*M* = 9.5, *SD* = 5.4) and the implementation intention condition (*M* = 9.1, *SD* = 3.8), *t*(58) = 0.39, *p* = 0.699 (Fig. 2A). Participants made generally few errors, with ring contacts occurring *M* = 6.3% of the time (*SD* = 9.98). However, a significant effect of Time-to-Failure reflects that the error rate increased over time, *F*(9, 522) = 2.43, *p* = 0.01, and a marginally significant Condition effect suggests that implementation intention participants tended to make more errors than goal intention participants, *F*(1, 58) = 3.08, *p* = 0.085 (Fig. 2B). No interaction between Condition and Time-to-Failure emerged, *p* = 0.664.

#### RPE and pain ratings

Significant main effects of Time-to-Failure emerged for both RPE, *F*(9, 382.16) = 365.49, *p* < 0.001, and pain ratings, *F*(9, 383.18) = 273.94, *p* < 0.001, indicating that sensations of effort and pain intensified over time (Fig. 2C,D). Other effects were not significant, *p*s > 0.17. In the final 10% of task performance, participants in the goal and implementation intention condition rated RPE with values of *M* = 8.2 (*SD* = 2.2) and *M* = 9.0 (*SD* = 2.0), and pain with values of *M* = 7.9 (*SD* = 2.2) and *M* = 8.5 (*SD* = 2.0), respectively. Values between 7 and 9 correspond to perceiving effort or pain as “very hard” to “extremely hard”, suggesting that participants were willing to exert themselves.

### Hemodynamic Data

Preliminary analyses revealed no baseline differences in O_2_Hb and HHb between conditions or between dorsal and ventral LPFC, *p*s > 0.48. We still specified baseline concentrations as covariate in the following analyses to account for the existing variance.

#### Oxygenated hemoglobin (O_2_Hb) concentration

**Figure 3:**
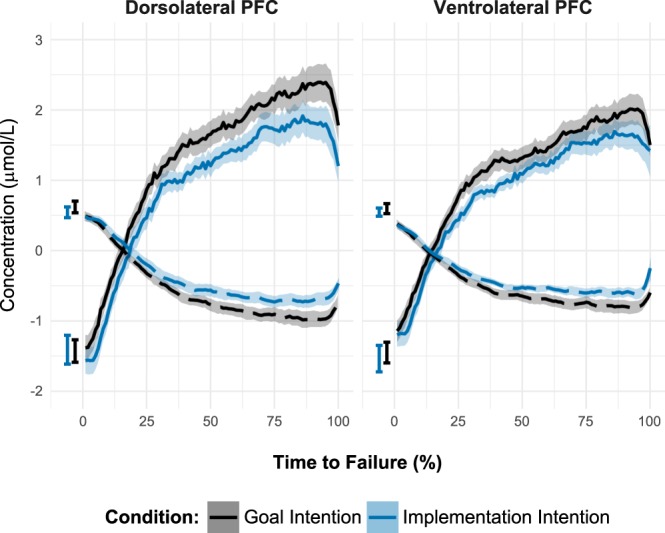
Changes in O_2_Hb (solid lines) and HHb (dashed lines) as a function of Time-to-Failure, Region of Interest, and Condition. Shaded regions represent standard errors at each 1% time-to-failure interval. Error bars represent average baseline values ± one standard error of the mean.

We found significant main effects of Time-to-Failure, *F*(9, 519.85) = 121.89, *p* < 0.001, ROI, *F*(1, 56.43) = 21.88, *p* < 0.001, and Condition, *F*(1, 57.15) = 4.91, *p* = 0.031, as well as a significant effect of the Baseline covariate, *F*(1, 112.49) = 22.91, *p* < 0.001. Moreover, significant interactions emerged between Time-to-Failure and ROI, *F*(9, 511.70) = 28.33, *p* < 0.001, and between ROI and Condition, *F*(1, 56.29) = 5.10, *p* = 0.028. Other effects were not significant, *p*s > 0.98. As illustrated in Fig. 3, the O_2_Hb concentration increased more strongly in the dorsal than in the ventral LPFC. Moreover, O_2_Hb concentration was lower among implementation intention than goal intention participants in the dorsal, *t*(61.71) = 2.61, *p* = 0.023, but not in the ventral LPFC, *t*(61.47) = 1.73, *p* = 0.174, suggesting that the self-regulation strategies differentially affected dorsal LPFC activation.

#### De-oxygenated hemoglobin (HHb) concentration

We found significant main effects of Time-to-Failure, *F*(9, 522.57) = 144.50, *p* < 0.001, and ROI, *F*(1, 52.45) = 4.92, *p* = 0.031, as well as a significant effect of the Baseline covariate, *F*(1, 114.00) = 17.05, *p* < 0.001. Moreover, a significant interaction between Time-to-Failure and ROI emerged, *F*(9, 514.98) = 36.85, *p* < 0.001. Other effects were not significant, *p*s > 0.12. As illustrated in Fig. 3, the HHb concentration decreased more strongly in the dorsal LPFC than in the ventral LPFC. Although differences between the implementation intention and the goal condition were in the same direction as for O_2_Hb concentration, they did not reach conventional levels of significance.

## Discussion

We found that oxygenation of the LPFC increases over the course of a physically exhausting task, which might indicate a steady increase of self-control demands due to a steady increase in perceptions of effort and pain. Forming implementation intentions did not alter perceptions of pain or effort during the task and failed to facilitate performance in terms of time-to-failure. On the cerebral level, the oxygenation in the LPFC was less pronounced in the implementation intention than the goal intention condition throughout the task, particularly in dorsal areas.

Taken together, our findings are consistent with the idea that self-regulatory processes are involved in strenuous physical exercise and corroborate existing research showing that increasing self-regulatory demands are mirrored by an increase in LPFC activation. Additionally, we observed that the self-regulation strategies of forming goal versus implementation intentions differentially affect cerebral activation patterns during exercise but not necessarily performance and perceptions of effort. This sheds a novel light on the role of self-regulation and of the LPFC for endurance performance.

### The LPFC During Physical Endurance

Corroborating existing research on cardiovascular endurance, we observed an increase in LPFC activation during the exercise^15^, ending in a plateau and a final drop immediately before task termination (Fig. 3). This is particularly noteworthy because the physiological demands imposed by static muscular endurance differ substantially from those in a cardiovascular endurance task: The HRT poses muscle fatigue as the primary challenge, whereas cardiovascular endurance is limited by anaerobic capacity^61^. Nevertheless, our results suggest that the cortical response is similar. One reason might be that the LPFC activity is associated with self-regulatory demands which are generally required in time-to-failure tasks: One has to keep going in spite of a steady increase in the perception of effort. This requires focused attention on the task at hand and the willingness to continuously invest effort and to resist the impulse to quit. In light of the LPFC’s role in self-regulation, our findings suggest that the increase, the plateau, and the subsequent drop in activation might reflect a more general neuronal mechanism behind increasing self-control demands.

The Expected Value of Control (EVC) theory^62^ offers a mechanistic explanation for this interpretation. Like performing physical tasks, exerting self-control is perceived as effortful^63^ and carries intrinsic costs that increase with control demands^64^ and that people try to avoid^65,66^. Whether or not (or how much) control is exerted depends on a cost-benefit analysis that maximizes the value of control^65^. EVC theory differentiates three components of control: Lower-order aspects of information processing (e.g., attention) need to be regulated (e.g., how much effort should be invested) and the corresponding regulative function (e.g., attentional control) must be specified and its execution monitored. The dorsal Anterior Cingulate Cortex (dACC) is assumed to compute the EVC of available control signals, choose the optimal signal, and relay this information to other brain structures like the LPFC^65^. Importantly, the dACC also specifies the intensity of the chosen control signal, thereby achieving a balance between automatic and controlled processing and minimizing control costs. The LPFC is then responsible for the effortful top-down regulation of the specified regulative function.

Our results can be tentatively interpreted from the perspective of EVC theory: The intensity of the control necessary for performing the static muscular endurance task increased over time, reflected in a steady increase in LPFC activation and RPE. Towards the end, the required control surpassed the EVC, rendering the ‘allowed’ intensity of the regulative effort insufficient for task continuation. In our data, this shows up as a plateau and an eventual drop in LPFC activation (i.e., a proxy for the intensity of the invested regulative effort), which ultimately became insufficient to regulate the high levels of perceived effort and pain (i.e., proxies for how much control was required). To directly test this explanation, future research should concurrently monitor dACC and PFC activity during straining physical exercise.

Taking evidence regarding the cell biology that underlies the hemodynamics of brain activity into account^67^, it is plausible that the increase in control signal strength primarily reflects increases in excitatory neurotransmission. In our data, the increase in O_2_Hb was accompanied by a comparatively smaller decline in HHb. Apparently, there is an increase in oxygenated blood that exceeds the rate of oxygen consumption. This might be interpreted as indirect evidence for an increase in excitatory neurotransmission as the task gets more demanding: Glutamate is the brain’s main excitatory neurotransmitter and is converted to Glutamine in an anaerobic process that uses glucose (glycolysis)^68^. To test this idea, future research might monitor brain oxygenation and glucose levels concurrently over the course of a fatiguing self-regulation task.

### Self-regulation Strategies

Contrary to our expectations, participants in the implementation intention condition did not outperform participants with a goal intention. If anything, they tended to report higher levels of pain and committed more errors. Although these trends did not reach statistical significance, they fit with previous findings in a similar setting^12^ (but see^69^, for facilitative effects of implementation intentions on endurance performance). However, forming implementation intentions was associated with a consistently lower activation of the LPFC throughout the task. Interestingly, implementation intentions do not seem to have uniformly modulated LPFC activity; instead, their effects were more pronounced in dorsal than in ventral portions of the LPFC. As the dorsolateral PFC is particularly related to focused attention and the ventrolateral PFC is associated more with behavioral control^19^, this suggests that implementation intentions reduce attention-related aspects of effortful regulation more effectively than inhibitory aspects. This is plausible given that the implementation intention used in the present study specified attentional aspects of endurance performance (i.e., ignoring sensations of pain). Moreover, it suggests that implementation intentions might have down-regulated effortful aspects of attentional control, which seem to be vital for persisting in a straining endurance task.

Corroborating previous findings (e.g.^39^), our results suggest that implementation intentions facilitate the automation of behavior as indicated by the reduced LPFC involvement. From the perspective of EVC theory, this finding might indicate that implementation intentions bias the EVC computation towards more automated processing, thereby relaying a less intense top-down control signal to the LPFC. As implementation intentions did not lead to improved performance one – somewhat provocative – interpretation of our findings might be that endurance performance does not necessarily benefit from a shift towards automatic processing. Indeed, endurance athletes have to constantly attend to inner and outer states to allow for successful goal striving^20^. Thus, reducing the capacity for controlled and effortful regulation might be detrimental for performance. Research on the cognitive strategies employed by athletes from different levels in training and in competition lends support to this idea^26^: Particularly in competition, world class runners prefer to closely attend to their internal sensations in order to be able to maximize their performance (i.e., they use associative cognitive strategies). This is in contrast to dissociative strategies (i.e., shunning sensory input from conscious awareness), which are preferred in training and often employed by less successful athletes^70^.

Encouragingly, this reasoning in turn suggests that implementation intentions specifying different critical cues and/or goal-directed behaviors might actually enhance endurance performance (e.g., resulting in an associative rather than a dissociative strategy). This would be consistent with research in other domains that demonstrates how strongly the selected cues and behaviors influence implementation intention effects (e.g.^41^). For example, antecedent-focused implementation intentions have been found to down-regulate the valence of negative emotions, whereas response-focused implementation intentions affect the associated arousal^71^. Implementation intentions can also be rendered more or less effective and susceptible to available resources depending on the specified cues and behaviors. For instance, planning concrete actions is more effective under cognitive strain (but less flexible) than planning to engage in certain thoughts^72^. Taken together, this suggests that future research may systematically investigate the effects of different types of implementation intentions on endurance performance.

### Limitations

The study results stem from a student sample with considerable variation in terms of sports engagement and prior experience. As we have argued above, self-regulation strategies like implementation intentions might differ between sport contexts and athletes. Accordingly, it might be that implementation intention effects evince in more homogeneous samples of athletes. Another possibility is that implementation intentions should be tailored more strongly on the individual level. Future research should directly investigate these possibilities, for example, by investigating the effect of implementation intentions in elite athletes. Moreover, although a goal intention condition is commonly used as control condition in implementation intention research^30^, it would still be interesting to add a further control condition to investigate whether any self-regulation strategy (goal or implementation intention) affects endurance performance in comparison to having no strategy.

We have interpreted our findings from a self-regulation perspective: Longer task duration led to higher self-regulatory demands which conversely resulted in an increase in LPFC activation. In line with the assumption that implementation intentions automate behavior^39^, we observed a less pronounced increase of LPFC activation in the implementation intention group. One might argue, however, that the increase in LPFC activation may also reflect the increase in RPE. Corroborating this interpretation, activation in PFC areas has been linked to both perceptions of effort and to the application of control^73,74^. To complicate matters further, a rise in RPE is directly linked to increased self-regulatory demands, as perceived exertion acts as an internal signal for the costs of an ongoing task^63,75^. Thus, continuing in spite of high costs requires more self-regulatory effort. Disentangling both concepts on a neural level is an important task for future research. Our data thus do not provide a definite answer to the question of whether LPFC activity reflects self-regulatory demands, RPE, or a combination of both. Yet, it should be noted that the group differences in LPFC activation were not observed in the RPE data. We are thus still inclined to interpret the rise in LPFC activation as primarily reflecting the application of control.

## Conclusion

Research on the limits of endurance performance has so far paid little attention to potential psychological factors and their neural mechanisms. In the present study, we hypothesized that people’s self-regulation of behavior is one such factor. In line with this assumption, we observed increasing activity of the LPFC during task performance with a sudden decrease immediately prior to task termination. Stronger activation of dorsal compared to ventral areas of the LPFC suggests that endurance performance might depend more strongly on self-regulatory functions associated with effortful attentional than on inhibitory control. Further, we found that the self-regulation strategy of forming implementation intentions reduced LPFC activation compared to goal intentions, with a stronger effect on dorsal than ventral areas. This is in line with prior research on implementation intentions and suggests automated action control. However, it also implies that implementation intentions might crowd out aspects of effortful control that seem vital in the context of maintaining a physically straining activity. In line with this assertion, implementation intentions failed to facilitate endurance performance. Taken together, the present research sheds new light on the role of self-regulation in endurance performance.

## Footnotes

1 We excluded the answer of one participant which was obviously not meant seriously: at a reported age of 41 years the participant indicated “walking” as main sport for the last 40 years.
